# Strength and Conditioning Practices and Perspectives of Volleyball Coaches and Players

**DOI:** 10.3390/sports9020028

**Published:** 2021-02-13

**Authors:** Anthony Weldon, Jason T. S. Mak, Sing T. Wong, Michael J. Duncan, Neil D. Clarke, Chris Bishop

**Affiliations:** 1 Faculty of Management and Hospitality, Department of Sports and Recreation, The Technological and Higher Education Institute of Hong Kong, Hong Kong, China; jasonm@vtc.edu.hk (J.T.S.M.); singwong@vtc.edu.hk (S.T.W.); 2 Centre for Sport, Exercise and Life Sciences, Coventry University, Coventry CV1 5FB, UK; aa8396@coventry.ac.uk (M.J.D.); ab1633@coventry.ac.uk (N.D.C.); 3 Faculty of Science and Technology, London Sports Institute, Middlesex University, London NW1 4RL, UK; c.bishop@mdx.ac.uk

**Keywords:** exercise selection, physical development, programing, sport, fitness, injury

## Abstract

To the authors’ knowledge this is the first study to describe the strength and conditioning (S&C) practices and perspectives of volleyball coaches and players. In total, 30 volleyball coaches (mean age 34.47 ± 7.83 years and coaching experience 19.57 ± 8.28 years), and 30 volleyball players (mean age 22.03 ± 4.43 years and playing experience 10.43 ± 8.98 years) completed an online survey with six sections: (a) informed consent; (b) background information; (c) education, qualifications, and prescription; (d) views on S&C; (e) exercise selection and preferences; and (f) issues and improvements. Frequency analysis was used to report responses to fixed-response questions and thematic-analysis for open-ended questions. While only one participant possessed an S&C certification, S&C was deemed ‘important’ to ‘very important’ for volleyball skills, physical fitness, and injury parameters. However, due to a reported lack of expertise, there appeared to be a theoretical understanding to practice gap. Furthermore, the implementation of S&C was considerably hindered by a lack of time, facilities, and equipment. National sports associations, coaches, and players can use the information within this study to provide an understanding of the current practices and perspectives of S&C in volleyball. While also promoting future developments in volleyball related S&C research and practice.

## 1. Introduction

Volleyball is a globally participated sport with 222 member federations and over 500 million registered players [1,2]. Playing positions include outside hitter, middle blocker, opposite, setter, libero, and defensive specialist [3]. The objective is to hit the ball over the net within the specified court dimensions and prevent the opposing team from returning the ball [3].

Beyond the technical and tactical requirements of volleyball, strength, power, and speed are considered the most significant factors determining competitive performance [4,5]. These physical attributes are required during rallies, which involve explosive movements such as spiking, blocking, and diving [3,6]. Work-to-rest ratios in high-level male volleyball players are approximately 1:6 (4.99 s of work to 29.02 s rest), therefore it is considered a high-intensity anaerobic sport [6,7,8].

To develop high-performing and robust volleyball athletes, strength and conditioning (S&C) programs with progressively overloaded whole-body exercises and position-specific movements are recommended [4]. It is important to concurrently develop general strength, power, and speed, to help underpin sport-specific training, performance, and injury reduction [4]. In particular, plyometrics are essential to volleyball training and can develop players’ performance in vertical and horizontal jumping, strength, flexibility, speed, and agility [8]. The prescription of plyometrics should be individualized for each athlete’s physical ability and playing position, as significant differences have been observed for upper body and lower body strength [9], lower body power, and sprint performance [10] across playing positions.

Repetitive jumping, landing, hitting, and blocking movements conducted in volleyball can increase injury prevalence of the ankles, knees, lower back, and shoulders [11,12,13]. However, volleyball is deemed relatively safe with an injury incidence of elite players during match play being 10.7/1000 h played, with 32.5% of all injuries causing subsequent time-loss 3.8/1000 h played, which is similar across club and collegiate levels [11]. Injury prevention measures are still of high importance to ensure players are physically fit and available for selection [11,13]. This can be generally achieved through manipulating training load and volume, improving strength (particularly eccentric) and muscular endurance [12,13]. For example, to reduce overuse injuries of the knees an individualized S&C program is recommended, incorporating conditioning exercises of the thigh, hip, and core muscles to efficiently absorb landing forces from repetitive jumps [12,13].

Although the significance of physical development in volleyball is undeniable, there is limited literature on the actual training practices in this sport [14]. Research suggests significant relationships (*p* ≤ 0.05) between structured S&C programs and volleyball position-specific game stats, including defensive specialists with squat and total strength; setters with hang cleans, T-drill, and broad jump; pin hitters with vertical jump, squat, and total strength; and middle blockers with broad jump [10]. However, not all teams have S&C coaches to design and implement their programs. Therefore, coaches, players, and support staff may be tasked with this responsibility.

Therefore, this study aims to assess the S&C practices and perspectives of volleyball coaches and players. To the authors’ knowledge, this is the first study to specifically address this. Results from this study will help ascertain how well coaches’ and players’ practices align with current research and guidelines in S&C. In turn, this study will support the development of S&C education, practice, and research in volleyball.

## 2. Materials and Methods

### 2.1. Experimental Approach to the Problem

This study used an anonymous online survey to investigate the S&C practices and perspectives of volleyball coaches and players. The survey was adapted from previous research [15,16,17] and developed using the open-access survey application Google Forms. All information within the survey was presented in English and Chinese for clarity and understanding. The survey comprised six sections: (a) written informed consent; (b) background information; (c) education, qualifications, and prescription; (d) views on S&C; (e) exercise selection and preferences; and (f) issues and improvements. The coaches survey included 25 fixed-responses and 25 open-ended questions, and the players’ survey included 24 fixed-responses and 25 open-ended questions (see Supplementary Appendix). The coaches’ additional question was regarding the age range of volleyball athletes coached. Some questions allowed the selection of multiple-responses, meaning some questions had more responses than others. Pilot testing was conducted by each member of the research team, three volleyball coaches, and three volleyball players, for three rounds before the survey was finalized. This led to slight modifications to the wording and structure of the survey to ensure its validity for use with this population.

### 2.2. Subjects

Overall, 30 volleyball coaches and 30 volleyball players participated in this study. All were registered under the Volleyball Association of Hong Kong, China Limited. The inclusion criteria for coaches were (1) a qualified volleyball coach, and (2) players coached currently perform resistance training practices. The inclusion criteria for players were (1) currently playing in competitive level volleyball, and (2) currently performing resistance training practices. All participants provided informed consent to initiate the survey. The survey started with an explanation of the purpose, aims, required time-commitment, and confidentiality of information.

### 2.3. Statistical Analyses

All responses from Google Forms were downloaded into a Microsoft Excel Spreadsheet. Fixed-response questions were assessed using frequency analysis. Open-ended response questions were assessed using thematic analysis [18], with the following six-stage process: (a) familiarization with the data; (b) generating initial codes; (c) searching for themes; (d) reviewing themes; (e) defining and naming themes; and (f) producing the report. This method of thematic analysis has been previously used in studies surveying S&C coaches [15,16,17,19,20]. Thereafter, overarching clear and identifiably distinct themes, representing the main ideas or patterns emerging from the raw data were generated for each question. Some responses provided sufficient information such that more than one overarching theme could be identified.

## 3. Results

### 3.1. Background Information

In total, 30 volleyball coaches (*n* = 21 male, *n* = 8 female, and *n* = 1 non-disclosed; mean age of 34.47 ± 7.83 years; mean coaching experience of 19.57 ± 8.28 years) and 30 volleyball players (*n* = 16 female, *n* = 14 male; mean age of 22.03 ± 4.43 years; mean playing experience of 10.43 ± 8.98 years) participated in this study.

The highest level of competition for coaches included Hong Kong Schools Sports Federation (HKSSF) (30%), Volleyball Association of Hong Kong, China Ltd. (VBAHK) League Division B/C (23%), VBAHK League Division A1/A2 (20%), Asian Volleyball Confederation (AVC) Competition (20%), Fédération Internationale de Volleyball (FIVB) International Competition (3%), and Other (3%). The highest level of competition for players included VBAHK League Division B/C (30%), AVC Competition (27%), VBAHK League Division A1/A2 (17%), University Sports Federation of Hong Kong, China Ltd. (USFHK) (17%), FIVB International Competition (10%).

The current roles of coaches consisted of head coach (73%), assistant coach (23%), and trainer (3%), and the age groups coached were 15–17 years (73.3%), above 18 years (47%), 12–14 years (40%), and below 12 years (20%). Players’ predominant positions were outside hitter (47%), setter (27%), opposite (10%), middle blocker (10%), and libero (7%).

### 3.2. Education, Qualifications, and Prescription

Coaches’ highest level of education was bachelor’s degree (47%), master’s degree (24%), higher diploma or associate degree (13%), and secondary school (17%), with 40% of qualifications being in a sports-related field. Players’ highest level of education was bachelor’s degree (50%), higher diploma or associate degree (47%), and secondary school (3%), with 53% of qualifications being in a sport-related field. Only one participant (a coach) (3%) held an S&C certification, which was with the United Kingdom Strength and Conditioning Association (UKSCA). Professional volleyball coaching certifications were held by 87% of coaches, with the following organizations and levels, VBAHK level one (43%), VBAHK level two (30%), FIVB level one (10%), FIVB level three (10%), and FIVB level two (7%). For players, 33% held coaching qualifications with the following organizations and levels, VBAHK level one (30%), and VBAHK level two (3%).

**Figure 1:**
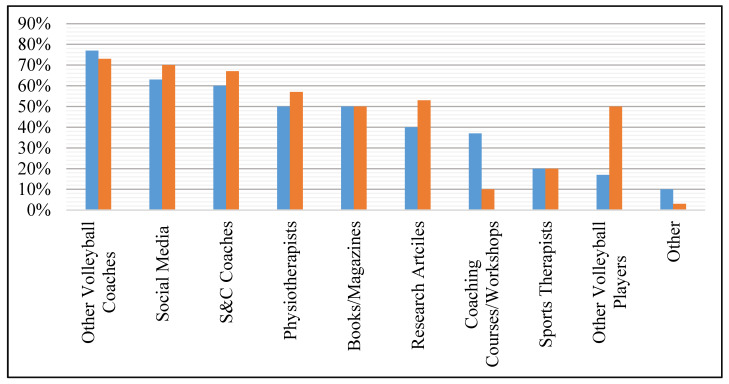
Percentage of coaches (blue) and players (orange) who obtain strength and conditioning information from different sources.

**Figure 2:**
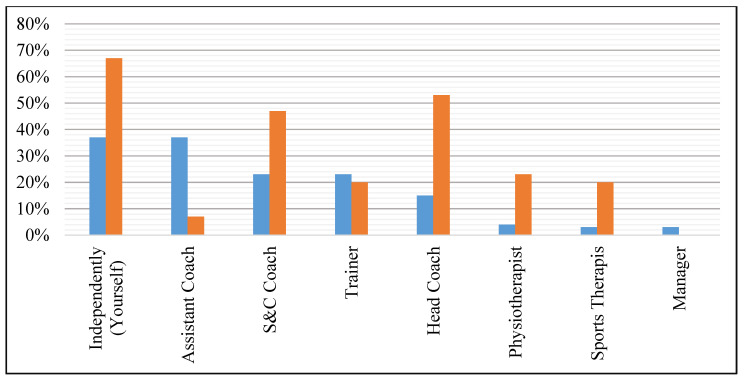
Percentage of coaches (blue) and players (orange) reporting the personnel responsible for prescribing strength and conditioning exercises.

The prevalence of different sources used by coaches and players for S&C information, and personnel responsible for prescribing S&C exercises is displayed in Figure 1 and Figure 2.

The prevalence of coaches and players perceived importance of S&C for different volleyball skills is displayed in Table 1, and for different physical, fitness, and injury parameters is displayed Table 2.

### 3.3. Views on Strength and Conditioning

The importance of S&C for different volleyball skills is presented in Table 3, and for different physical, fitness, and injury parameters is presented in Table 4.

Coaches reported the effectiveness of their current S&C programs used with players to be moderately effective (53%), effective (23%), slightly effective (13%), and very effective (10%). Players reported the effectiveness of their current S&C programs to be effective (43%), moderately effective (33%), very effective (20%), and slightly effective (3%). Other responses from coaches included “insufficient training sessions and knowledge on fitness training”, “players fitness levels cannot be enhanced/maintained without professional and systematic training arrangements”, “training mainly focuses on bodyweight without training equipment and due to the busy school/work schedule the training time is insufficient in Hong Kong compared to other countries. Therefore, physical improvements are non-significant”, “lack of S&C knowledge”, “players with high attendance showed improvements”. Other responses from players included “I am not sure what is meant by effective. I have strength improvements, but I still feel pain in my previously injured shoulder”, “no S&C coach available for planning fitness training. Our coach mainly focuses on ball practice”, “reaction time is faster and strength has improved”, “I continued my S&C training during the pandemic. Even with little volleyball training, I was able to maintain my performance”, “it helps improve my performance and prevent injuries during training (e.g., maintains spiking power and endurance), which lowers my chance of injury when tired”.

### 3.4. Exercise Selection and Preferences

The exercise preferences of coaches and players to develop different physical and volleyball-specific attributes, and to decrease the likelihood of injuries are presented in Table 5.

### 3.5. Issues and Improvements

The different issues, disadvantages and desired improvements coaches and players reported in regards to delivering S&C programs are presented in Table 6.

## 4. Discussion

To the authors’ knowledge, this is the first study to address the practices and perspectives of volleyball coaches and players regarding S&C. A key finding from this survey is that coaches and players are the main individuals responsible for prescribing their S&C training (see Figure 2). Furthermore, coaches and players predominantly source their S&C information from peers within volleyball (e.g., head coach) (see Figure 1). However, this is problematic given that out of all participants, only one coach had an official S&C qualification and there was limited formal education in sports-related fields. Coaches and players mostly declared that S&C was required to develop volleyball skills, physical, fitness, and injury parameters, emphasizing the demand for quality S&C training in volleyball. However, in Hong Kong there were a reported lack of S&C coaches available to work specifically in volleyball or the available finances to employ an S&C coach. Furthermore, other limitations, such as facilities, equipment and time, added to the difficulty in providing high-quality S&C provisions. This was further supported by only 10–20% of participants believing their current S&C practices were ‘very effective’, suggesting room for improvement.

Most coaches and players considered S&C training ‘important’ to ‘very important’ for all volleyball skills (see Table 1). For attacking based movements, spiking was seen as an area in which S&C could develop performance most, with power and speed being the primary objectives (see Table 3). Research in elite female volleyball players has demonstrated that a tailored S&C program can improve upper body strength (e.g., bench press, pullover) and power (e.g., medicine ball overhead throw) over a season [21]. However, these physical improvements were not transferred to volleyball-skill performance (i.e., jump spike speed) [21]. The variable most correlated with jump spike speed was standing spike speed, suggesting the importance of movement and velocity specificity when developing sports skills, which is sometimes difficult to achieve with S&C exercises [4,21,22]. This notion is supported through literature investigating the effects of an 8-week training program, consisting of three skill-based court sessions per week on skill and physical fitness parameters in volleyball players [23]. Results indicated no significant improvements in lower-body and upper-body muscular power, but spiking skill performance significantly improved, which included criteria of speed, accuracy, and technical competency [23]. For defensive based movements, blocking was considered an area where S&C could improve performance most, with jumping and blocking height the primary objectives (see Table 3). To improve jumping height, a key focus of S&C training in volleyball is likely to be plyometrics [8]. A recent systematic review including 15 studies demonstrated that plyometric training increases vertical and horizontal jump performance in volleyball players [8]. More specifically, two studies included in this review showed that a structured plyometric training program specifically improves two-foot, right-foot, left-foot, and side-step block jumps [24,25].

Almost all coaches and players believed S&C to be ‘important’ to ‘very important’ for developing strength, power, and speed (see Table 2). It is well established within the literature that an S&C program with an initial focus on strength development before training more power-based exercises (e.g., jumping) is beneficial in improving athletic ability [26]. To program and individualize such training within volleyball it has been suggested to develop a force-velocity profile of each player [27]. However, further evidence on how this approach influences the physical development of players and positively transfers to volleyball performance is required [27]. Encouragingly, it was also reported that S&C was considered ‘important’ to ‘very important’ for reducing injuries, rehabilitating injuries, and supporting players in their return to play (see Table 2). The most prevalent injuries in volleyball are acute ankle injuries and overuse injuries of the knee and shoulder [13,28]. A key area for reducing the likelihood of such injuries is to systematically monitor and modify volleyball training load and volume, which is a common role for S&C coaches, and ensures training and match-play stimuli are suitable for players [13]. Some coaches and players suggested S&C may lead to overtraining, fatigue, and increase injury prevalence (see Table 5). However, it is believed these participants considered S&C as an additional workload to existing skill-based training, as opposed to modifying workload to include S&C. To reduce volleyball-related injuries, players are encouraged to undertake a year-round S&C program, with a primary focus on eccentric exercises of the rotator cuff muscles, core strengthening, and stability training of the ankles, knees, and shoulders [13]. Accordingly, coaches and players also reported that strength and stability development were the primary means of S&C to reduce injuries (see Table 4).

For strength development, injury reduction, and volleyball performance, the most preferred exercises used by coaches and players was the squat and associated variations (see Table 5). This is similar to that reported by S&C coaches in other sports for strength and power development [15,16,17,29,30,31,32,33,34]. As various postures and movements in volleyball require the adoption of a squat position (e.g., digging), it is highly recommended that players perform the squat, including associated variations (e.g., front squat, back squat, goblet squat), to improve strength and power capabilities [6]. Furthermore, it has been shown, in non-elite female volleyball players, that one-repetition maximum squat strength relative to body weight is very strongly correlated (*r* = 0.95) with vertical jump height, which has a positive transfer to different volleyball movements (e.g., blocking) [35]. The rationale of coaches and players for prescribing the squat was to develop lower body strength, jumping, and landing ability (i.e., stronger and more stable ankles, knees, and hips), which is in line with the aforementioned research recommendations. Coaches and players reported that plyometrics was the most commonly used exercise to develop speed, power, and volleyball-specific fitness (see Table 5). This again is similar to that prescribed by S&C coaches in other sports [15,16,17,29,30,31,32,33,34] and supported by contemporary research in volleyball, which suggests implementing plyometric exercises used for speed and power development into sport-specific practice [36]. The combination of plyometrics and skill-based volleyball activity has shown to simultaneously improve jumping ability and volleyball skills (e.g., throwing) [36]. However, the magnitude of improvement must consider the players’ level. For example, if the player is elite and has high-level technical skills, it is likely the greatest improvements will be related to power and speed, as opposed to skill performance [36].

The main issues faced by coaches and players in this study regarding the implementation of S&C, were a lack of time, facilities, equipment, and S&C expertise available in Hong Kong (see Table 6). This demonstrates that S&C could be more comprehensive and widespread if local infrastructures enabled this in a more formalized manner. However, this is not uncommon even in professional sport. For example, strength and conditioning coaches working in cricket and soccer across various countries also stated that time was the biggest issue faced within their role, with a lack of facilities and equipment also being commonly reported [16,17]. In regards to expertise, there is limited formal or professional education and qualifications available in Hong Kong, which is a possible reason for the lack of practitioners. For example, only one coach held an S&C certification which was from the UK Strength and Conditioning Association (UKSCA). With this lack of education and perceived ability to prescribe S&C practices in mind, it is not entirely surprising the main disadvantage of S&C perceived by coaches was the potential to increase injury risk. Contrary to the beliefs of these coaches, studies have elucidated that when implemented appropriately, conducting strength training is safe [37,38]. It was reassuring to see that some coaches and players reported they wanted to develop their S&C knowledge and practical ability through further education and consultation with S&C professionals (see Table 5). The area that coaches and players perceived the greatest potential for improvement was periodization. This is in line with the reported time constraints and lack of access to facilities. Therefore making better use of the available time may be a viable strategy for greater integration of S&C into volleyball. Research suggests that programs adhering to strategic periodization strategies compared to non-periodized programs are superior for strength and power development, which are key components of volleyball performance [39,40].

## 5. Practical Applications

Coaches and players perceived S&C to be highly important for improving volleyball performance and reducing injuries. However, it was difficult to implement high-quality S&C practices, due to a lack of relevant expertise, time, facilities, and equipment. Although opportunities for professional development in S&C and particularly S&C in volleyball are limited in Hong Kong, coaches and players are advised to undertake related education and accreditations if possible. Alternatively, teams may consult with or employ a qualified S&C coach. This highlights the importance of professional S&C organizations providing overseas education and accreditation programs, to upskill and qualify those responsible for S&C delivery.

## 6. Limitations

This study surveyed 30 volleyball coaches and 30 players, and although this sample size is reasonable for this level of coach and player in Hong Kong, it may not be representative of all coaches and players within this demographic or extrapolated to those in other countries. Furthermore, due to the sample size, a sub-analysis comparing coaches and players across different ages or levels of competition was not possible. The required number of surveys required for valid analysis was not determined before data collection. However, it was aimed to obtain as many responses as possible within this purposive sample of coaches and players.

## Figures and Tables

**Table 1:** The importance of strength and conditioning training for volleyball skills reported by coaches and players.

		Not Important (%)	Slightly Important (%)	Moderately Important (%)	Important (%)	Very Important (%)
Spiking	Coach	3	0	3	27	67
	Player	0	0	0	30	70
Blocking	Coach	0	3	0	33	63
	Player	0	0	0	30	70
Serving	Coach	0	0	10	47	43
	Player	0	0	17	43	40
Defending	Coach	0	3	3	23	70
	Player	0	0	0	47	53
Setting	Coach	0	3	7	33	57
	Player	0	3	7	47	43
Mean	Coach	0	1.8	4.6	32.6	60
	Player	0	0.6	4.8	39.4	55.2

**Table 2:** The importance of strength and conditioning training for different physical, fitness and injury parameters, reported by coaches and players.

		Not Important (%)	Slightly Important (%)	Moderately Important (%)	Important (%)	Very Important (%)
Strength	Coach	0	0	3.3	23.3	73.3
	Player	0	3.3	0	23.3	73.3
Power/Speed	Coach	0	0	0	20	80
	Player	0	0	0	23	77
Volleyball-Specific Fitness	Coach	0	0	3	27	70
	Player	0	0	0	27	73
Reducing Injuries	Coach	0	0	0	23	77
	Player	0	0	7	20	73
Injury Rehabilitation/ Return to Play	Coach	3.3	0	3.3	30	63.3
	Player	0	0	3.3	33.3	63.3
Mean	Coach	0.6	0.0	1.8	24.6	72.6
	Player	0.0	0.6	2.0	25.2	71.8

**Table 3:** Relative importance of strength and conditioning for different volleyball skills, reported by coaches and players.

Volleyball Skill	Rank	Theme	Exemplar Response	Percentage Coaches	Percentage Players	Percentage Difference
Spiking	1	Power/Speed	“Spiking requires power generated from the shoulder, forearm, and core”	60	67	−7
	2	Injury Reduction	“S&C helps decrease injuries when doing repetitive spike training”	30	27	3
	3	Strength	“Increased strength improves spiking ability”	30	23	7
	4	Jumping/Landing	“S&C training improves jumping ability for a higher spiking point”	23	20	3
	5	Coordination/Movement Quality	“Specific S&C can develop spiking movement and quality”	20	10	10
	6	Fitness/Endurance	“Good fitness and endurance enables high quality repeated jumps and spikes”	3	7	−4
	7	Miscellaneous	“Enhances the performance”	0	10	−10
Blocking	1	Jumping/Blocking Height	“Blocking height is determined by jumping ability, which can be improved with S&C”	53	43	10
	2	Speed/Power	“Develops movement speed and hangtime”	37	27	10
	3	Postural Control/Stiff Block	“Being able to perform a stiff block with good posture”	30	37	−7
	4	Injury Reduction	“Decreases injuries associated with muscle weakness or soreness”	27	3	24
	5	Strength	“Strength is the foundation for effective jumping and blocking”	13	37	−24
	6	Balance	“Helps keep balance while in the air”	10	0	10
	7	Agility	“Improves reaction time and agility”	7	0	7
	8	Fitness/Endurance	“Competing and blocking requires a certain level of fitness”	3	7	−4
Serving	1	Hitting Power	“The faster the arm swing the more powerful the serve”	60	60	0
	2	Injury Reduction	“Reduce the risk of shoulder injuries”	33	3	30
	3	Strength	“With the trend of jump serves, muscle strength plays an important role in smashing the ball”	30	3	27
	4	Coordination/Movement Quality	“Assists with body coordination especially for jump serves”	23	47	−24
	5	Jump Height	“Jumping power is needed to increase the height of jump serve”	23	3	20
	6	Fitness/Endurance	“Improved fitness can help stabilize performance in the latter part of competition”	10	0	10
	7	Miscellaneous	“Controlling the technique of service is more important than muscle strength”	3	0	3
	8	Core	“Core muscles are required to stabilize the body”	0	10	−10
Defending	1	Reaction/Movement Speed	“Helps develop quickness and reaction speed which is important in defence”	60	47	13
	2	Fitness/Endurance	“Need to squat a long time so endurance in this position is key”	23	23	0
	3	Injury Reduction	“Due to high moving speed, S&C helps prevent injuries”	23	0	23
	4	Strength	“Volleyball defensive positions require high quadriceps and hamstrings muscle strength”	17	13	4
	5	Power	“S&C helps players possess enough power to defend effectively in games”	10	27	−17
	6	Miscellaneous	“Determining the defensive area”	7	0	7
	7	Flexibility/Mobility	“Mobility to get into defensive postures”	3	7	−4
Setting	1	Reaction/Movement Speed	“Setting from your teammate requires a quick response and fast movement”	37	13	24
	2	Stability	“Improves the stability of the jump set”	33	30	3
	3	Strength	“Strengthening the legs, core and arms is good for setting”	30	10	20
	4	Injury Reduction	“Decreases injury occurrence”	23	0	23
	5	Jumping	“Develops jumping ability for the jump set”	10	23	−13
	6	Fitness/Endurance	“Adequate cardiorespiratory fitness is required for repeated setting during competition”	7	0	7
	7	Miscellaneous	“To adapt to different tactics”	7	10	−3
	8	Balance/Coordination	“Helps with body balance during jump setting”	0	23	−23
	9	Power	“Power in the arms is needed to set the ball outside the hitter’s spot”	0	17	−17

Some answers detailed more than one response, which was further sub-divided amongst the themes created.

**Table 4:** Perceived benefits of strength and conditioning for training different physical fitness and injury parameters, reported by coaches and players.

Physical, Fitness, and Injury Parameters	Rank	Theme	Exemplar Response	Percentage Coaches	Percentage Players	Percentage Difference
Strength	1	Improve Volleyball Performance	“Muscle strength is fundamental for high performing players and winning competitions”	33	17	16
	2	Strength	“Weight training is required to develop players strength”	30	47	−17
	3	Injury Reduction	“Reduces the risk of acute and chronic injuries”	27	0	27
	4	Power	“Power comes from a basis of strength, therefore needs to be trained”	13	37	−24
	5	Coordination/ Movement Quality	“Players can learn correct movement techniques and improve coordination on the court”	7	0	7
	6	Fitness	“Maintain enough fitness for competition”	7	7	0
	7	Miscellaneous	“The two are correlated”	7	3	4
Speed/ Power	1	Power/Speed	“Power is essential as players need to suddenly jump or move quickly to react”	57	63	−6
	2	Improve Volleyball Performance	“Improves volleyball performance in serving and spiking”	23	20	3
	3	Movement/Coordination	“Enhances muscle coordination, and the correct contraction/relaxation of muscles”	23	13	10
	4	Injury Reduction	“Correct technique when conducting power exercises will help reduce injuries”	20	0	20
	5	Strength	“Strong muscles are required to support fast movements”	13	13	0
	6	Fitness	“Fitness training can incorporate speed and power work”	3	7	−4
	7	Miscellaneous	“Developing speed and power is important”	3	10	−7
Volleyball-Specific Fitness	1	Specific Movement Development	“S&C improves jumping, running and arm swinging which are volleyball-specific movements”	37	27	10
	2	Improve Volleyball Performance	“Train specific muscles to enhance volleyball performance”	33	30	3
	3	Fitness	“Greater fitness increases the chances of winning”	20	0	20
	4	Injury Reduction	“Develop physical ability to reduce injuries”	13	0	13
	5	Positional Improvements	“Can specifically train the characteristics of different positions”	13	3	10
	6	Power/Speed	“Helps achieve higher, stronger and faster volleyball athletes”	10	40	−30
	7	Miscellaneous	“In Hong Kong, we have limited time to conduct S&C, due to court availability”	7	7	0
	8	Strength	“Muscular strength supports different types of movement, such as sudden changes of direction in competition”	7	10	−3
Injury Reduction	1	Strength	“Strengthening muscles supports the body through protecting joints”	60	27	33
	2	Stability/Postural Control	“S&C training can help enhance balance and motor control”	20	30	−10
	3	Injury Reduction	“Gets rid of injuries”	13	7	6
	4	Flexibility/Mobility	“Improves flexibility, which allows the joints to perform different movements”	10	0	10
	5	Fitness	“Adequate fitness levels are needed to perform skills and movements freely, and for preventing injuries”	7	23	−16
	6	Individualized Programming	“S&C can discover individual differences, which can be trained”	3	0	3
	7	Power	“Prevents problems, like the use of power in wrong postures”	3	10	−7
	8	Miscellaneous	“Injury is the enemy of athletes”	0	7	−7
Injury Rehabilitation/ Return to Play	1	Enhanced Recovery	“S&C training can speed up recovery and help players adapt to training demands”	47	43	4
	2	Strength	“Muscles atrophy and regress during injury, therefore need to be strengthened”	33	47	−14
	3	Decrease Re-Injury Risk	“It is easy to get injured once returning to training, so S&C helps overcome this”	23	10	13
	4	Accelerate Return to Play	“The most important thing for players is the time to get back on the court. The knowledge and technology nowadays speeds up this process”	20	17	3
	5	Flexibility/Mobility	“Provides an opportunity to improve flexibility that may have been a cause of injury”	6	7	−1
	6	Miscellaneous	“Depends on the level of injury”	0	7	−7

Some answers detailed more than one response, which was further sub-divided amongst the themes created.

**Table 5:** Preferred exercises of coaches and players for developing different physical, fitness and injury parameters.

Areas for Exercise Selection	Most Important Exercise	Exemplar Rationale for Exercise Selection	Percentage Coaches	Percentage Players	Percentage Difference
Strength	Squat and Variations	“Squatting increases leg muscle strength, leading to improved jumping and explosiveness”	52	60	−8
	Bench Press/Push Up	“Shoulder strength for volleyball performance improvement”	11	8	3
	Lunge and Variations	“Strengthening the leg muscles”	11	4	7
	Core (e.g., Plank)	“Whole-body is connected to the core, which affects movement coordination”	7	12	−5
	Miscellaneous (e.g., Interval Training)	“Interval training can cover different muscles simultaneously”	7	4	3
	Other (e.g., Burpee)	“Burpees train both strength and coordination”	7	4	3
	Pull Up	“A multi-joint exercise that increases upper body muscle strength”	4	8	−4
Speed/ Power	Plyometrics (e.g., Continuous Jumping)	“Ability to produce explosive force in a short amount of time”	36	37	−1
	Sprint/Run	“Improve running performance over specific distances covered on the court”	25	7	18
	Burpees	“Involves most muscle groups in the body”	11	4	7
	Agility Ladder	“Can work a combination of speed and agility”	7	0	7
	Core (e.g., Cable Trunk Rotation)	“Abdomen is one of the most important muscle groups to train for volleyball”	7	4	3
	Miscellaneous (e.g., Timed Training)	“Timed training can simulate game situations”	7	11	−4
	Bench Press/Push Up	“Effective upper body power training”	4	11	−8
	Other (e.g., Deadlift)	“Deadlifts can focus on training power of the legs and back”	4	4	0
	Squat	“Similar movement to jumping which requires power”	0	11	−11
	Olympic Weightlifting	“Can load and improve the power of specific movements related to volleyball”	0	11	−11
Volleyball-Specific Fitness	Plyometrics (e.g., Medicine Ball Slam)	“Enhances movement speed and jumping ability”	29	29	0
	Squat	“Helpful for defensive postures and jumping ability”	18	14	4
	Core (e.g., Deadbug)	“Core is the central area for power production”	14	25	−11
	Other (e.g., Side Lunge)	“Side lunge action is similar to underhand pass”	11	4	7
	Miscellaneous (e.g., Weight Training)	“Weight training is required to make athletes strong and powerful”	11	7	4
	Volleyball Movement	“Lateral movement to ball receive can train the whole body and help physical fitness”	7	11	−4
	Bench Press/Push Up	“Strengthen the upper body, especially the shoulders”	7	7	0
	Deadlift	“Covers a lot of muscle groups useful for volleyball”	4	4	0
Injury Reduction	Squat and Variations	“Reduces knee and ligament injuries”	38	19	20
	Flexibility/Mobility (e.g., Stretching)	“Improves joint range of motion and ability of muscle extension”	19	0	19
	Miscellaneous (e.g., Muscular Endurance)	“Muscle endurance is necessary. As we cannot determine the length of the game. Players need long-lasting physical ability”	19	3	16
	Core (e.g., Side Plank)	“Core exercises improve your balance and stability, which is important for reducing injuries”	12	19	−7
	Plyometrics (e.g., Box Landing)	“Improves how people land, which is how a lot of injuries occur”	8	37	−29
	Other (e.g., Nordics)	“Nordics help make the hamstrings resilient to injury”	4	14	−11
	Push Up	“Works the shoulders and arms, which are used a lot in volleyball”	0	8	−8
Volleyball Performance	Squat and Variations)	“Strengthens and supports joints for landing”	36	44	−8
	Plyometrics (e.g., Dumbbell Jump)	“Vertical jumping is required for blocking, serving, and spiking”	28	22	6
	Core (e.g., Plank)	“Core exercises like the plank can train the whole body”	12	11	1
	Other (e.g., Shuttle Runs)	“Shuttle runs are very important for chasing and going after the ball”	12	4	8
	Miscellaneous (e.g., Muscular Endurance)	“Volleyball players require repeated power, so muscular endurance should be trained”	8	0	8
	Deadlift	“All round exercise that should be performed”	4	4	0
	Push Up	“Important for spiking action”	0	7	−7
	Olympic Weightlifting	“Can develop an athlete to jump and land, while improving spiking and vertical blocking performance”	0	7	−7

**Table 6:** Coaches and players reported issues, disadvantages and desired improvements when implementing strength and conditioning.

	Rank	Theme	Exemplar Response	Percentage Coaches	Percentage Players	Percentage Difference
Issues	1	Time	“There is no regular physical training time”	60	20	40
	2	Facilities	“Very little facilities and space available”	30	13	17
	3	Equipment	“Lack of equipment in our venue”	17	20	−3
	4	Expertise	“We have limited knowledge regarding which exercises to do and how to do them”	17	30	−13
	5	Miscellaneous	“Difficult to maximize power in the gym room by yourself”	13	23	−10
Disadvantages	1	Increased Injury Risk	“Actions demonstrated maybe incorrect leading to more injuries”	27	7	20
	2	None	n/a	27	20	7
	3	Miscellaneous	“It is difficult to determine the upper limit of athletes”	23	17	7
	4	Overtraining/Fatigue	“Unsure about how much training and recovery is required”	20	10	10
	5	Enjoyment	“Generally muscular strength and physical training will be dull”	10	27	−17
	6	Lack of Expertise	“It requires assistance from professionals and hiring an S&C coach is expensive”	0	7	−7
Desired Improvements	1	Periodization	“Specify the correct number of hours for the periodic physical training plan each week, including the exercises”	30	77	−47
	2	Education/Consultation	“Enrol on S&C courses”	20	13	7
	3	Individualized Programming	“Before training perform detailed assessments of players, so programs can be created”	17	3	13
	4	Miscellaneous	“More sleep and eat together at lunchtime”	17	10	7
	5	S&C within Volleyball Training	“Integrate S&C into volleyball technical training”	10	0	10
	6	Flexibility/Mobility	“More warm-up, cool down and flexibility training”	0	10	−10

## Data Availability

Data sharing is not applicable to this article.

## Supplementary Materials

Digital copies of the surveys used in this study can be found online at https://www.mdpi.com/2075-4663/9/2/28/s1.
