# Supplementary material for: Strength and Conditioning Practices and Perspectives of Volleyball Coaches and Players
Source: Sports (Basel). 2021 Feb 13;9(2):28. doi: 10.3390/sports9020028 (PMC7917682; doi:10.3390/sports9020028)
Supplement: Supplementary file 1 [file sports-09-00028-s001.pdf]

sports

Supplementary Appendix (As Digital Content)

Coach Survey

- ▪ Refers to questions with multiple choice answers
- • Refers to questions with single choice answers

A) Informed Consent

- Agree
- Disagree

B) Background Information

Q1. Gender?

- Male
- Female
- Prefer not to say

Q2. How old are you? (must be over 18 years old to enter this survey)

Q3. Number of years' experience as a volleyball coach?

Q4. What is the highest level of competition that you have coached?

- Fédération Internationale de Volleyball (FIVB) International Competition
- Asian Volleyball Confederation (AVC) Competition
- Volleyball Association of Hong Kong, China Ltd (VBAHK) League (Division A1/A2)
- Volleyball Association of Hong Kong, China Ltd (VBAHK) League (Division B/C)
- The University Sports Federation of Hong Kong, China Ltd (USFHK) Competition
- The Hong Kong Schools Sports Federation (HKSSF) Competition
- Other: ______

Q5. Refer to Q4, what was your role?

- Head Coach
- Assistant Coach
- Trainer
- Other: ______

Q6. What is the current level of competition that you coach?

- Fédération Internationale de Volleyball (FIVB) International Competition
- Asian Volleyball Confederation (AVC) Competition
- Volleyball Association of Hong Kong, China Ltd (VBAHK) League (Division A1/A2)
- Volleyball Association of Hong Kong, China Ltd (VBAHK) League (Division B/C)

- The University Sports Federation of Hong Kong, China Ltd (USFHK) Competition
- The Hong Kong Schools Sports Federation (HKSSF) Competition
- Other: ______

Q7. Refer to Q6, what is your role?

- Head Coach
- Assistant Coach
- Trainer
- Other: ______

Q8. What age range do you predominantly coach?

- Below 12 years old
- 12-14 years old
- 15-17 years old
- Above 18 years old

C) Education, Qualifications and Prescription

Q9. What is your highest level of education?

- Secondary School
- Higher Diploma/Associate Degree
- Bachelor's Degree
- Master's degree
- Doctor of Philosophy (PhD)
- Other

Q10. What was the subject area of your highest level of education?

Q11. Do you possess a strength and conditioning qualification with any of the below organizations or any other related fitness qualification?

- Australian Strength and Conditioning Association (ASCA)
- National Strength and Conditioning Association (NSCA)
- Collegiate Strength and Conditioning Coaches Association (CSCCa)
- United Kingdom Strength and Conditioning Association (UKSCA)
- None
- Other

Q12. What professional volleyball coaching qualification(s) do you hold?

- Volleyball Association of Hong Kong, China Ltd (VBAHK) Level I
- Volleyball Association of Hong Kong, China Ltd (VBAHK) Level II
- Fédération Internationale de Volleyball (FIVB) International Level I
- Fédération Internationale de Volleyball (FIVB) II

- Fédération Internationale de Volleyball (FIVB) III
- None
- Other

Q13. Where do you predominantly source strength and conditioning information?

- Volleyball Coaches
- Volleyball Players
- Strength and Conditioning Coaches
- Sports Therapists
- Physiotherapists
- Coaching Courses/Workshops
- Research Articles
- Social Media
- Books and Magazines
- Other

Q14. Who is responsible for prescribing strength and conditioning exercises for your players?

- Manager
- Head Coach
- Assistant Coach
- Independently (Yourself)
- Trainer
- Strength and Conditioning Coach
- Sports Therapist
- Physiotherapist
- Other: ______

D) Views on Strength and Conditioning

Q15. How important is strength and conditioning for **volleyball spiking**?

- Not Important
- Slightly Important
- Moderately Important
- Important
- Very Important

Q15a. Refer to your previous answer, please explain why?

- ___________

Q16. How important is strength and conditioning for **volleyball blocking**?

- Not Important
- Slightly Important

- Moderately Important
- Important
- Very Important

Q16a. Refer to your previous answer, please explain why?

- ___________

Q17. How important is strength and conditioning for **volleyball serving**?

- Not Important
- Slightly Important
- Moderately Important
- Important
- Very Important

Q17a. Refer to your previous answer, please explain why?

- ___________

Q18. How important is strength and conditioning for **volleyball defending**?

- Not Important
- Slightly Important
- Moderately Important
- Important
- Very Important

Q18a. Refer to your previous answer, please explain why?

- ___________

Q19. How important is strength and conditioning for **volleyball setting**?

- Not Important
- Slightly Important
- Moderately Important
- Important
- Very Important

Q19a. Refer to your previous answer, please explain why?

- ___________

Q20. How important is strength and conditioning for **strength development**?

- Not Important
- Slightly Important
- Moderately Important
- Important

- Very Important

Q20a. Refer to your previous answer, please explain why?

- ___________

Q21. How important is strength and conditioning for **speed and power development**?

- Not Important
- Slightly Important
- Moderately Important
- Important
- Very Important

Q21a. Refer to your previous answer, please explain why?

- ___________

Q22. How important is strength and conditioning for **volleyball-specific fitness**?

- Not Important
- Slightly Important
- Moderately Important
- Important
- Very Important

Q22a. Refer to your previous answer, please explain why?

- ___________

Q23. How important is strength and conditioning for **reducing the likelihood of injuries**?

- Not Important
- Slightly Important
- Moderately Important
- Important
- Very Important

Q23a. Refer to your previous answer, please explain why?

- ___________

Q24. How important is strength and conditioning for **injury rehabilitation/return to play**?

- Not Important
- Slightly Important
- Moderately Important
- Important
- Very Important

Q24a. Refer to your previous answer, please explain why?

- ___________

Q25. How effective is your current strength and conditioning program?

- Not Effective
- Slightly Effective
- Moderately Effective
- Effective
- Very Effective

Q25a. Refer to your previous answer, please explain why?

- ___________

Q26. Do you have any other views on strength and conditioning in volleyball?

- ___________

<u>E) Exercise Selection</u>

Q27. Name up to '**THREE**' strength and conditioning exercises **in order** of importance**,** you consider beneficial for **<u>strength development</u>**?

1.
2.
3.

Q27a. Explain why you have listed the **'FIRST'** exercise most important?

Q28a. Name up to '**THREE**' strength and conditioning exercises **in order,** you consider beneficial for **<u>speed and power development</u>**?

1.
2.
3.

Q28a. Explain why you have listed the **'FIRST'** exercise most important?

Q29. Name up to '**THREE**' strength and conditioning exercises **in order,** you consider beneficial for **<u>volleyball-specific fitness</u>**?

1.
2.
3.

Q29a. Explain why you have listed the **'FIRST'** exercise most important?

Q30. Name up to 'THREE' strength and conditioning exercises **in order,** you consider beneficial for **reducing the likelihood of volleyball related injuries**?

1.
2.
3.

Q30a. Explain why you have listed the **'FIRST'** exercise most important?

Q31. Name the top '**THREE'** strength and conditioning exercises **in order,** you consider beneficial for **volleyball performance**?

1.
2.
3.

Q31a. Explain why you have listed the **'FIRST'** exercise most important?

F) Issues and Improvements

Q32. Explain what the biggest issues you face when implementing strength and conditioning?

Q33. Explain any disadvantages associated with strength and conditioning?

Q34. Given unlimited time and resources, how would you change or improve your current strength and conditioning provisions?

Player Survey

- ▪ Refers to questions with multiple choice answers
- • Refers to questions with single choice answers

A) Informed Consent

- Agree
- Disagree

B) Background Information

Q1. Gender?

- Male
- Female
- Prefer not to say

Q2. How old are you? (must be over 18 years old to enter this survey)

Q3. Number of years' experience as a volleyball player?

Q4. What is the highest level of competition that you have played in?

- Fédération Internationale de Volleyball (FIVB) International Competition
- Asian Volleyball Confederation (AVC) Competition
- Volleyball Association of Hong Kong, China Ltd (VBAHK) League (Division A1/A2)
- Volleyball Association of Hong Kong, China Ltd (VBAHK) League (Division B/C)
- The University Sports Federation of Hong Kong, China Ltd (USFHK) Competition
- The Hong Kong Schools Sports Federation (HKSSF) Competition
- Other: ______

Q5. Refer to Q4, what was your predominant position?

- Outside Hitter
- Middle Blocker
- Opposite
- Setter
- Libero
- Defensive Specialist
- Other: ______

Q6. What is your current level of competition that you play in?

- Fédération Internationale de Volleyball (FIVB) International Competition
- Asian Volleyball Confederation (AVC) Competition
- Volleyball Association of Hong Kong, China Ltd (VBAHK) League (Division A1/A2)
- Volleyball Association of Hong Kong, China Ltd (VBAHK) League (Division B/C)
- The University Sports Federation of Hong Kong, China Ltd (USFHK) Competition
- The Hong Kong Schools Sports Federation (HKSSF) Competition
- Other: ______

Q7. Refer to Q6, what is your predominant position?

- Outside Hitter
- Middle Blocker
- Opposite
- Setter
- Libero
- Defensive Specialist
- Other: ______

C) Education, Qualifications and Prescription

Q8. What is your highest level of education?

- Secondary School
- Higher Diploma/Associate Degree
- Bachelor's Degree
- Master's degree
- Doctor of Philosophy (PhD)
- Other

Q9. What was the subject area of your highest level of education?

Q10. Do you possess a strength and conditioning qualification with any of the below organizations or any other related fitness qualification?

- Australian Strength and Conditioning Association (ASCA)
- National Strength and Conditioning Association (NSCA)
- Collegiate Strength and Conditioning Coaches Association (CSCCa)
- United Kingdom Strength and Conditioning Association (UKSCA)
- None
- Other

Q11. What professional volleyball coaching qualification(s) do you hold?

- Volleyball Association of Hong Kong, China Ltd (VBAHK) Level I
- Volleyball Association of Hong Kong, China Ltd (VBAHK) Level II
- Fédération Internationale de Volleyball (FIVB) International Level I
- Fédération Internationale de Volleyball (FIVB) II
- Fédération Internationale de Volleyball (FIVB) III
- None
- Other

Q12. Where do you predominantly source strength and conditioning information?

- Volleyball Coaches

- Volleyball Players
- Strength and Conditioning Coaches
- Sports Therapists
- Physiotherapists
- Coaching Courses
- Research Articles
- Social Media
- Books and Magazines
- Other

Q13. Who is responsible for prescribing strength and conditioning exercises for you?

- Manager
- Head Coach
- Assistant Coach
- Independently (Yourself)
- Trainer
- Strength and Conditioning Coach
- Sports Therapist
- Physiotherapist
- Other: ______

D) Views on Strength and Conditioning

Q14. How important is strength and conditioning for **volleyball spiking**?

- Not Important
- Slightly Important
- Moderately Important
- Important
- Very Important

Q14a. Refer to your previous answer, please explain why?

- ___________

Q15. How important is strength and conditioning for **volleyball blocking**?

- Not Important
- Slightly Important
- Moderately Important
- Important
- Very Important

Q15a. Refer to your previous answer, please explain why?

- ___________

Q16. How important is strength and conditioning for **volleyball serving**?

- Not Important
- Slightly Important
- Moderately Important
- Important
- Very Important

Q16a. Refer to your previous answer, please explain why?

- __________

Q17. How important is strength and conditioning for **volleyball defending**?

- Not Important
- Slightly Important
- Moderately Important
- Important
- Very Important

Q17a. Refer to your previous answer, please explain why?

- __________

Q18. How important is strength and conditioning for **volleyball setting**?

- Not Important
- Slightly Important
- Moderately Important
- Important
- Very Important

Q18a. Refer to your previous answer, please explain why?

- __________

Q19. How important is strength and conditioning for **strength development**?

- Not Important
- Slightly Important
- Moderately Important
- Important
- Very Important

Q19a. Refer to your previous answer, please explain why?

- __________

Q20. How important is strength and conditioning for **speed and power development**?

- Not Important
- Slightly Important
- Moderately Important
- Important
- Very Important

Q20a. Refer to your previous answer, please explain why?

- ____________

Q21. How important is strength and conditioning for **volleyball-specific fitness**?

- Not Important
- Slightly Important
- Moderately Important
- Important
- Very Important

Q21a. Refer to your previous answer, please explain why?

- ____________

Q22. How important is strength and conditioning for **reducing the likelihood of injuries**?

- Not Important
- Slightly Important
- Moderately Important
- Important
- Very Important

Q22a. Refer to your previous answer, please explain why?

- ____________

Q23. How important is strength and conditioning for **injury rehabilitation/return to play**?

- Not Important
- Slightly Important
- Moderately Important
- Important
- Very Important

Q23a. Refer to your previous answer, please explain why?

- ____________

Q24. How effective is your current strength and conditioning program?

- Not Effective
- Slightly Effective

- Moderately Effective
- Effective
- Very Effective

Q24a. Refer to your previous answer, please explain why?

- ___________

Q25. Do you have any other views on strength and conditioning in volleyball?

- ___________

<u>E) Exercise Selection</u>

Q26. Name up to '**THREE**' strength and conditioning exercises **in order,** you consider most important for <u>**strength development**</u>?

1.
2.
3.

Q26a. Explain why you have listed the **'FIRST'** exercise most important?

Q27a. Name up to '**THREE**' strength and conditioning exercises **in order,** you consider most important for <u>**speed and power development**</u>?

1.
2.
3.

Q27a. Explain why you have listed the **'FIRST'** exercise most important?

Q28. Name up to '**THREE**' strength and conditioning exercises **in order,** you consider most important for <u>**volleyball-specific fitness**</u>?

1.
2.
3.

Q28a. Explain why you have listed the **'FIRST'** exercise most important?

Q29. Name up to '**THREE**' strength and conditioning exercises **in order,** you consider most important for <u>**reducing the likelihood of volleyball related injuries**</u>?

1.
2.
3.

Q29a. Explain why you have listed the **'FIRST'** exercise most important?

Q30. Name up to '**THREE'** strength and conditioning exercises **in order,** you consider most important for **<u>volleyball performance</u>**?

1.
2.
3.

Q30a. Explain why you have listed the **'FIRST'** exercise most important?

<u>F) Issues and Improvements</u>

Q31. Explain the biggest issues you face when implementing strength and conditioning?

Q32. Explain any disadvantages associated with strength and conditioning?

Q33. Given unlimited time and resources, how would you change or improve your current strength and conditioning provisions?
